# The Body Wall Microbiome of the Terrestrial Slug *Deroceras laeve* Reveals Potential Endosymbionts and Shares Core Organisms with Other Mollusks

**DOI:** 10.1007/s00248-025-02652-8

**Published:** 2025-11-18

**Authors:** Wilbert Gutiérrez-Sarmiento, Mayela Fosado-Mendoza, Carlos Lozano-Flores, Alfredo Varela-Echavarría

**Affiliations:** Instituto de Neurobiología, Universidad Nacional Autónoma de México (UNAM), Querétaro, México

**Keywords:** Shotgun metagenomics, Archaeome, Bacteriome, Mycobiome, Virome

## Abstract

The marsh slug *Deroceras laeve* is an invasive mollusk found in gardens, field crops, and wetlands. It lacks a protective shell, suggesting that microbial communities are associated with its adaptability to the environment. Here, we used a whole shotgun metagenomic approach to analyse the complex microbiome of *D. laeve* and compared it to that of other mollusks. This demonstrated the presence in *D. laeve* of bacteriophages such as *Erwinia phage*, *Certrevirus*, and *Machinavirus*, which target plant pathogen bacteria. In the Archaea domain the halophilics *Halovivax* and *Halobaculum* predominated, but also present were the methanogens *Methanobacterium*, *Methanobrevibacter*, *Methanocaldococcus*, *Methanococcus*, and *Methanosarcina*, involved in phosphate solubilization and methanogenesis during decomposition of organic matter. The Bacteria domain was dominated by γ-Pseudomonadota such as *Buttiauxella*, *Citrobacter*, *Enterobacter*, *Klebsiella*, *Kluyvera*, *Leclercia*, and *Pseudomonas* which are producers of enzymes that degrade biomass and complex carbohydrates. Regarding the fungal community, filamentous or yeast ascomycetes predominated such as *Debaryomyces*, *Puccina*, and *Pyricularia* known as plant pathogens or associated with decaying organic matter. Consistent with these findings, functional analysis revealed enrichment in genes involved in fermentation and carbohydrate metabolism. Remarkably, regardless of species, ecosystem, and tissue type, we found that the core microbiome of the mollusks in this study is mainly structured by the Phyla Uroviricota, Euryarchaeaota, Pseudomonadota, and Ascomycota, with diversity at the genus level. This suggests ancient symbiotic interactions of these mollusks with specific types of microbes which may have been critical for adaptability to their environment.

**Supplementary Information:**

The online version contains supplementary material available at 10.1007/s00248-025-02652-8.

## Introduction

The microbial communities associated with animals, whether commensal, pathogenic or symbiotic, are collectively defined as the microbiota. At a higher complexity level, the genomes of the microbiota and their functional interaction with the host and its environment constitute the microbiome which plays a central role in diverse aspects such as environmental adaptability, functional profiling and metabolic regulation [1]. Of great relevance in this context is the “core microbiota” which is the set of microbial taxa that are consistently shared by a group of animals thereby shaping their ecology and evolution [2].

Studies of microbiomes employ a variety of tools including microbial isolation, metabolomics, and various high-throughput DNA sequencing approaches [3]. Among them, metabacording is widely used as it allows studies of archaea, bacteria and fungi, although it often lacks species resolution. Transcriptomics affords complementary insights into active microbial functions but requiring specific measures to prevent RNA degradation, while shotgun metagenomic approaches as used in this study provide a comprehensive overview of the microbiome albeit entailing a computationally intensive process.

The microbiome of mollusks, particularly gastropods, is of great interest due to their exceptional morphological and ecological diversity. This group, which includes terrestrial snails and slugs, exhibits a variety of life forms and adaptations that make them intriguing models. Since gastropods have colonized a variety of habitats, including freshwater, marine, and terrestrial ecosystems, their microbiota most likely plays a significant part in their ability to adapt to these diverse environments [4]. Therefore, understanding their mostly unexplored relationship with their associated microorganisms might reveal insights into adaptative mechanisms critical to their survival.

Research on the microbiome of marine slugs has revealed complex interactions essential for processes such as cellulose metabolism and the promotion of immune status in *Haliotis tuberculata* and *Haliotis diversicolor* [5]. Carbohydrate metabolism and antagonism against pathogens have been linked to the microbiome of the freshwater snails *Biomphalaria straminea* and *Biomphalaria glabrata* [6]. The antagonistic role of the microbiome against pathogens has also been addressed in the terrestrial slugs *Deroceras reticulatum* and *Ambigolimax valentianus* [7]. Even the cultivable cellulolytic bacteria of the gastrointestinal tract of the snail *Achatina fulica* has been described [8]. Terrestrial slugs, such as members of the genus *Deroceras*, have also been studied to unravel their microbiome and its influencing factors [9, 10].

Land slugs are of significant relevance because their lack of protective shells exposes them directly to diverse environmental threats and pathogens, suggesting that they employ unique means of symbiotic or defensive mechanisms for protection and survival in cosmopolitan habitats. The integumentum or body wall of gastropods is the outermost barrier in direct contact with the environment. It likely hosts a rich diversity of microbes that may perform essential functions maintaining host homeostasis which has not been studied thus far.

**Fig. 1:**
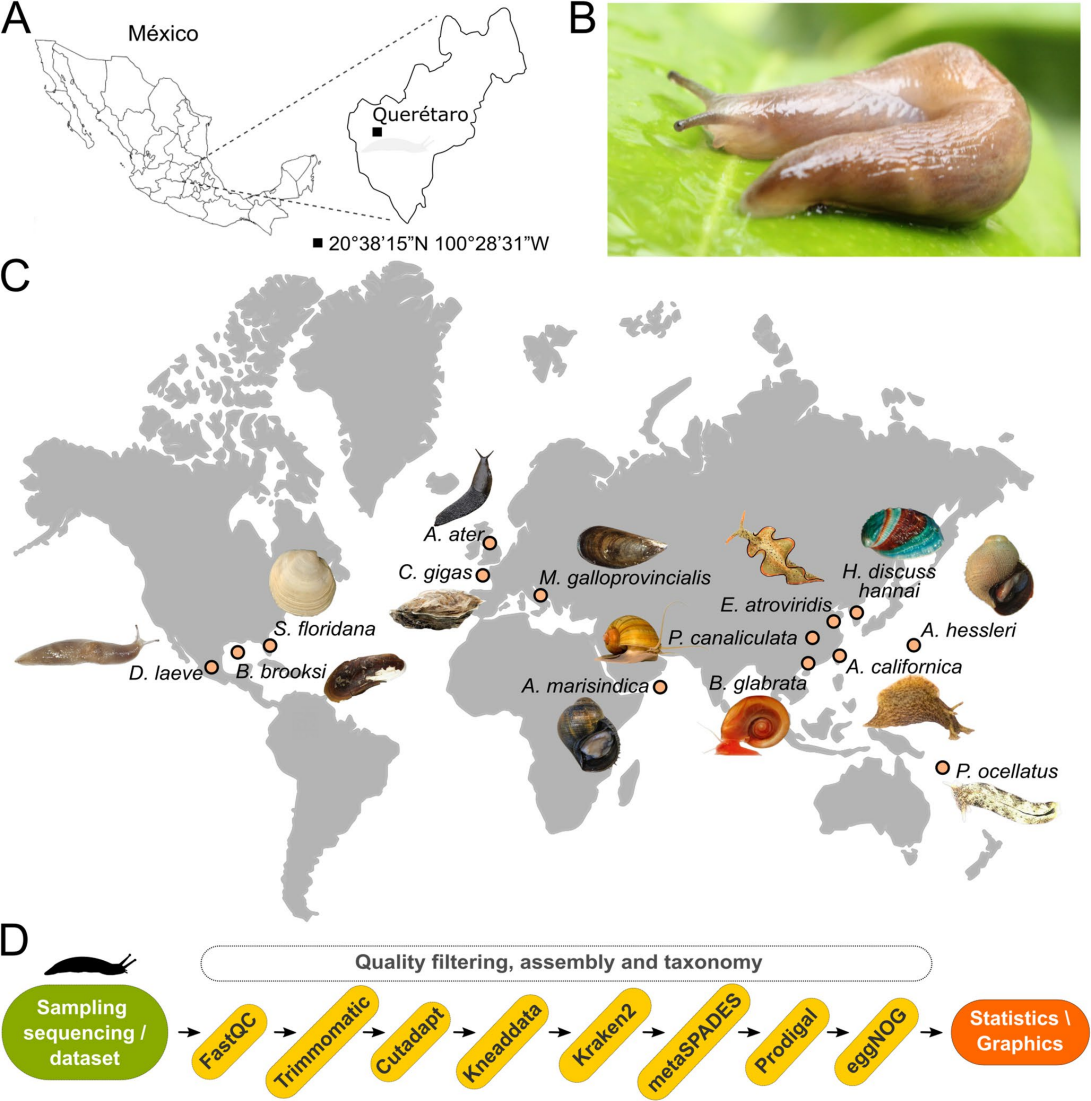
The land slug *Deroceras laeve*. **A**) Specimens were collected at an urban garden in Querétaro, México (20°38’15”N, 100°28’31”W) and kept under controlled laboratory conditions (18 °C, 12 h light/dark cycle). **B**) Wild *D. laeve* as observed in gardens. **C**). Geographic distribution of the 14 different mollusk species analyzed by a shotgun metagenomic approach. **D**). Full bioinformatic analysis workflow including sampling/DNA-Illumina sequencing, quality filtering (FastQC, Trimmomatic, Cutadapt, Kneaddata), taxonomy classification (Kraken2), assembly (metaSPADES), functional assignment (Prodigal and eggNOG), statistics, and plotting

In this work, we studied the microbiome of the globally dispersed marsh slug *Deroceras laeve* (Fig. 1A-B). The interest in this species stems from its potential as an experimental model to study regeneration, functional genomics, and genomic evolution. As part of our efforts to develop *D. laeve* as a laboratory model, we recently published SlugAtlas (https://slugatlas.lavis.unam.mx), an online anatomical and histological resource, along with a description of the intriguing aspects of *D. laeve* such as its capacity for degrowth-regrowth and regeneration [11]. We also sequenced and annotated its genome [12].

We assessed the microbiome from the body wall of lab-reared *D. laeve* with a metagenomic shotgun approach and compared it with those of other mollusks to explore how host phylogeny and living environment impact their composition. This work offers new perspectives on the co-evolution between gastropods and their associated microorganisms, while also revealing new microbial functions with potential biotechnological or medical applications.

## Materials and methods

### Study Design and Shotgun Metagenomic Analysis

In this study, we generated and analyzed a shotgun metagenomic dataset from the body wall of the marsh slug *D. laeve* (Fig. 1A-B) and compared it with shotgun metagenomes of thirteen other terrestrial, freshwater and seawater mollusk species from diverse world regions [57–63] (Fig. 1C and Supplemental File 1 - Table S1).

Specimens of *D. laeve* originally captured from gardens in Querétaro, México (20°38’15”N, 100°28’31”W) were reared in the laboratory for a year under controlled conditions (18 °C and a dark-light cycle of 12 h–12 h) at the Instituto de Neurobiología (INB) of the Universidad Nacional Autónoma de México (UNAM) (strain INB-UNAM [11]),. Five individuals, each three months old, weighing between 150 and 200 mg, were used for metagenomic analysis. Each specimen was processed independently, resulting in five distinct biological replicates. The animals were first anesthetized by immersion in 5% ethanol for 10 min, then the mucus covering the body was removed by rolling the animal on dry tissue paper followed by an additional rinse in 5% ethanol and dissection in water. The entire visceral mass, mantle complex, and head were removed and the body wall was rinsed again in water. DNA was extracted from the body wall with partial depletion of the host cells. The tissue was cut into small pieces with a razor blade, incubated for 1 h with slow rotation at 37 °C in 5 ml of Dulbecco´s Modified Eagle medium (DMEM, Invitrogen, USA) supplemented with 20 µg/ml of collagenase (17102-013, Gibco-BRL, USA) and 20 µg/ml of dispase (17105-041, Gibco-BRL, USA), and dissociated by pipetting up and down using a wide-bore pipette. Fresh DMEM (25 ml) was added, along with 150 µl of 10% Triton X-100, gently mixed by inversion for 2 min and centrifuged at 1509 *xg* for five minutes. The pellet was resuspended in Solution I of the DNA Power Soil Kit (47014, QIAGEN, Germany) and the DNA was extracted according to the manufacturer’s recommended protocol and resuspended in DNase/RNase-free water. DNA concentration was determined with the NanoDrop 1000 spectrophotometer (Thermo Scientific, USA) and DNA integrity was corroborated by electrophoresis on 1% agarose gel stained with GelRed (Biotium, CA, USA). Shotgun libraries were prepared with the Nextera DNA Flex Library Prep kit (Illumina) and sequenced using the Illumina NextSeq 500 platform (150 nucleotide paired-end reads) (Secuenciación y Bioinformática SENASICA, Tecámac, México). Raw data can be found in the NCBI SRA BioProject PRJNA1035784 (samples BC59-BC63). The sequences of the cytochrome oxidase 1 (COX1) coding region of all mollusk species used in this study were obtained from GenBank (Supplemental File 1 - Table S2) and multiple alignment was performed using the muscle tool (v3.8.31) with default settings. An unrooted phylogenetic tree was constructed using the Neighbor Joining (NJ) algorithm and the Jukes-Cantor distance measure, with 1000 bootstrap replicates performed in CLC Sequence Viewer (v8.0, Qiagen, Germany).

### Bioinformatic Analysis

The bioinformatic pipeline employed for this study (Fig. 1D) was carried out using the high-performance computing cluster of the Laboratorio Nacional de Visualización Científica Avanzada (LAVIS-SECIHTI, UNAM (https://www.lavis.unam.mx). We outlined within the GitHub repository https://github.com/WilbertGtzS/metagenomic_shotgun. FastQC (v0.11.3) was used for quality control (https://www.bioinformatics.babraham.ac.uk/projects/fastqc/). Trimmomatic (v0.39) was employed to eliminate sequence reads smaller than 50 base pairs and those with phred-based quality score lower than 25 [13], and Cutadapt (v4.0) was used to remove low quality 5’ and 3’ read ends (https://cutadapt.readthedocs.io/en/stable/index.html). Kneaddata (v0.12.3, https://huttenhower.sph.harvard.edu/kneaddata/) was used for decontaminating human sequences. High-quality reads were filtered to remove protozoa, plasmid, plant, and human sequences, and then assigned to archaea, bacteria, virus, and fungal taxonomy using Kraken2 (v2.1.3) with Plus PFP database (release 1/12/2024) [14]. For bacteria the updated nomenclature of Oren and Garrity [15] was employed. We applied quality control to remove spurious or low-confidence taxonomic assignments representing less than 0.01% of the total assigned reads per sample. The assembly of metagenomes was performed with metaSPAdes (v3.15.2) with default parameters of *metaspades* and *metaviralspades* modes [16]. The protein-encoding genes were predicted using Prodigal (v2.6.3.) [17] and the functional assignment was performed with eggNOG-mapper (v2.1.13) [18] with v2.1.9 database.

### Graphics and Statistical Analysis

All metagenomic sequences available for each mollusk species were treated as biological replicates and no technical replicates were included in this analysis. For comparative visualization, data were averaged and represented as a single bar or box per species in the plots. Rarefaction graphs were constructed in RStudio (4.2.2) using the normalization by *rrarefy* function with *vegan* package (2.6–4.6). Chao1 index (related to the number of different species) and the Shannon index (related to the relative abundance of each species) were calculated and plotted using *vegan* (2.6–4.6) and *ggplot2* (3.4.4) in RStudio (v4.2.2). The output raw counts of Kraken2 were normalized with the trimmed mean of M-values (TMM) method using edgeR (v4.2.2) and only taxa with more than 10 counts were analyzed further. The results of taxonomy assignment were visualized in Sankey diagrams with Pavian (v.1.2.1, https://github.com/fbreitwieser/pavian). Relative abundance bar plots between gastropod species were constructed in *phyloseq* (1.46.0) and *ggplot2* (3.4.4) in RStudio (4.2.2). Beta diversity was assessed using Non-metric Multidimensional Scaling (NMDS) implemented in the *phyloseq* package (v1.46.0). Differences in community composition between groups were tested using permutational multivariate analysis of variance (PERMANOVA) with 999 permutations, considering p-values < 0.05 as significant. To further identify which tissue types or ecosystems contributed most to the observed differences, pairwise PERMANOVA tests were performed with p-value adjustment for multiple comparisons using the Benjamini-Hochberg false discovery rate (FDR) correction. Additionally, differences in relative abundance were assessed with the nonparametric Kruskal-Wallis test followed by Dunn’s posthoc test for multiple comparisons, implemented with the *ggbetweenstats* function of the *ggstatsplot* package (v0.12.5). Sample sizes used in each comparison (metagenomic replicates) are indicated in Supplemental File 1 - Table S1 and were also incorporated into all statistical tests. The heatmap and barplot of eggNOG-functionality were performed with *dplyr*,* tidyr*,* GO.db*, *pheatmap* and *ggplot2* libraries on RStudio (4.2.2).

## Results

In this study we analysed the microbiome of the body wall of the marsh slug *D. laeve* and of thirteen other mollusk species including terrestrial, freshwater, and marine species (Fig. 1C and Supplemental File 1 - Table S1). For *D. laeve* we additionally assessed the microbial functional gene enrichment to ascertain biological processes that could be relevant for the interaction with the host.

Phylogenetic analysis of all the species in this study based on COX-1 sequence demonstrated that they belong to three major branches: (1) Heterobranchia with species from terrestrial, freshwater, and seawater habitats, (2) Caenogastropoda/Vetigastropoda including only species from freshwater and seawater habitats, and (3) Autobranchia with a single seawater species (Supplemental File 1 – Fig. S1). This provides context to assess how evolutionary history and ecological adaptability influence the molluscan microbiome.

For the analysis of the microbiota of *D. laeve* we employed a shotgun metagenomic approach which yielded over 12.4 millions of reads (M) of raw data, 11 M of which were quality reads. The metagenomes of the other thirteen mollusk species encompassed a total of 833.5 M of sequence data.

The taxonomy classification of the microbiota of *D. laeve* revealed a complex structure composed by 65 phyla, 150 classes, 326 orders, 845 families and 3101 genera. From the total count, this represents 73% of bacteria, 6.8% of fungi, 2.1% of archaea, and 1.6% of viruses (Supplemental File 1 – Fig. S2). Microbiome of the other species revealed all clear divergences between them and *D. laeve* but, surprisingly, also striking similarities which are described in the following sections.

### Rarefaction and Alpha Diversity

The rarefaction curves generated with the taxonomy classification results for viruses, archaea, bacteria, and fungi of *D. laeve* reached clear plateaus, indicating satisfactory identification coverage (Supplemental File 1- Fig. S3). The other species also reached saturation for archaea and fungi, while the curves for viruses and bacteria had an ascending component, suggesting that deeper sequencing could reveal additional species richness.

**Fig. 2:**
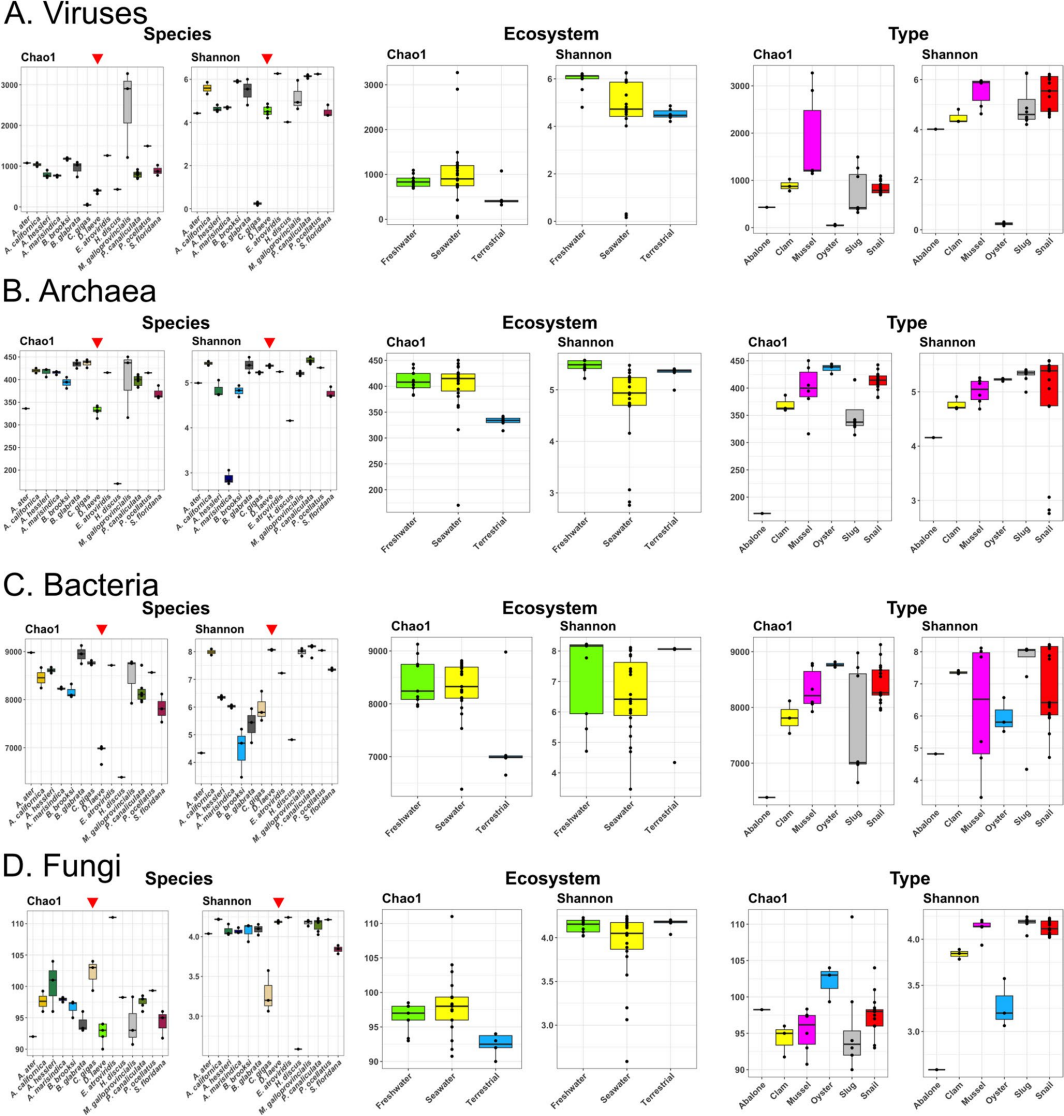
Alpha diversity estimators (Chao1 and Shannon) of microbial species found in *Deroceras laeve* and other mollusks based on the taxonomy assignment by Kraken2. Point charts are shown for (**A**) Viruses, (**B**) Archaea, (**C**) Bacteria, and (**D**) Fungi. Indexes of the left pair of panels were obtained based on the microbiome composition of individual species (*D. laeve* in each panel is indicated by a red triangle). The central pair of panels was based on the composition by ecosystem. In the right pair of panels, microbiomes were grouped by type of species

Alpha diversity analysis (Fig. 2) showed that bacterial communities had the highest Chao1 and Shannon indices, followed by viruses, archaea, and fungi. The *D. laeve* microbiome exhibited lower Chao1 richness across all microbial domains compared to most other mollusks, suggesting fewer rare species, potentially due to its rearing in lab conditions, in contrast to the wild environments of the other species. By ecosystem (Fig. 2 – central panels), terrestrial mollusks had significantly lower Chao1 values than freshwater and seawater species, indicating fewer rare taxa. However, Shannon diversity for archaea and fungi was higher in freshwater (*B. glabrata*, *P. canaliculata*) and terrestrial (*D. laeve*, *A. ater*) groups, suggesting more even and rich communities, in contrast to lower values in seawater mollusks. In terms of mollusk type (Fig. 2 – right panels), the abalone (*H. discus*) showed significantly lower Chao1 richness and Shannon diversity for viruses, archaea, and bacteria, indicating domination by few taxa. Similarly, the oyster (*C. gigas*) exhibited low fungal and viral diversity. In contrast, slugs, snails, clams, and mussels displayed a more balanced microbial community composition.

### Global Taxonomy of the Mollusk Microbiota

#### Viruses

**Fig. 3:**
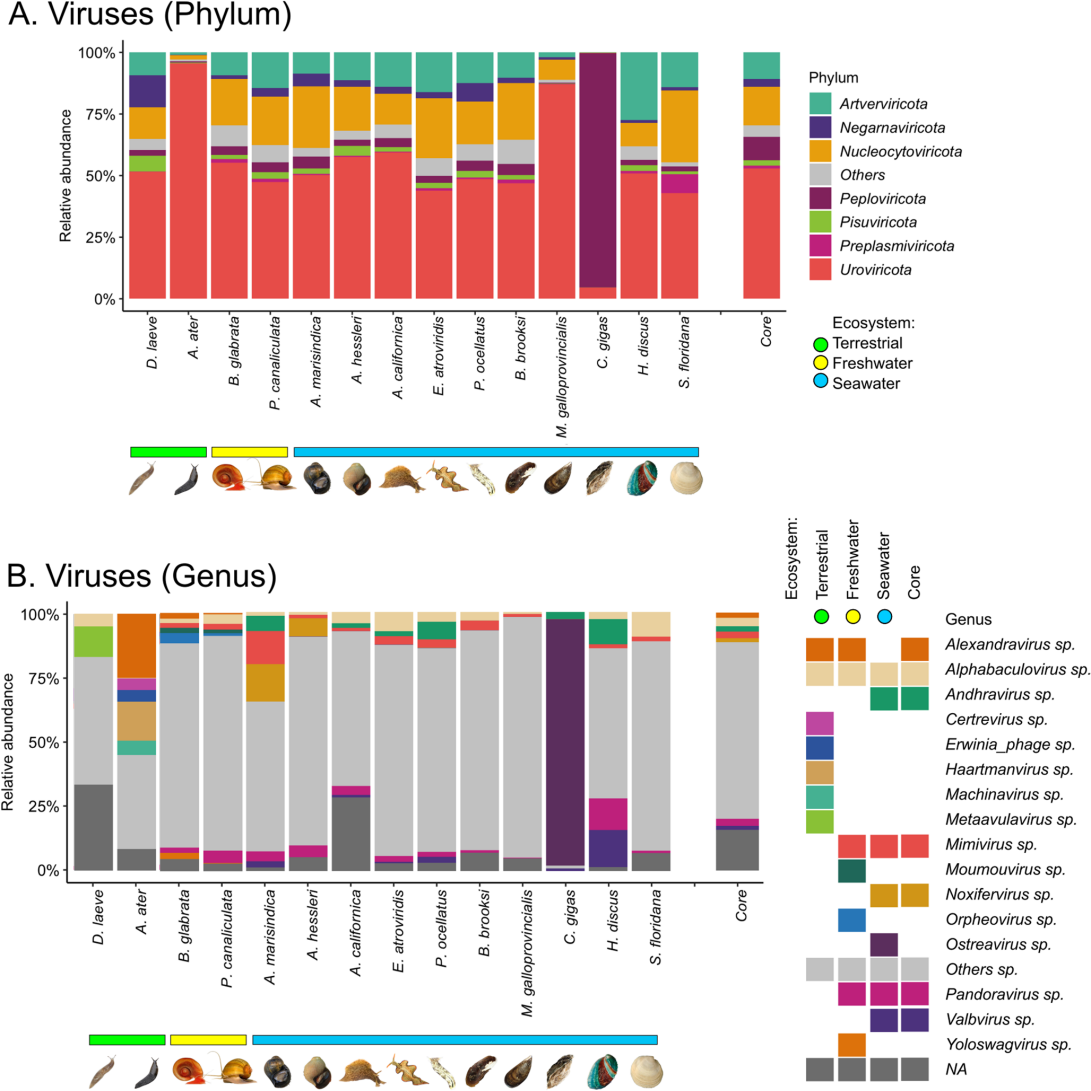
Comparison of the relative abundance of Viruses in the microbiome of *Deroceras laeve* and other mollusks analyzed by shotgun metagenome sequencing. Mollusk species are sorted by the ecosystem occupied by each along the X-axis: terrestrial (green horizontal bar), freshwater (yellow horizontal bar), and seawater (blue horizontal bar). (**A**) Barplot of the top seven phyla and “Others” based on relative abundance. (**B**) Barplot of relative abundance of sixteen predominant genera, “Others”, and “NA” (not assigned). On both, A and B, the last barplot shows the core composition of Viruses. The legend shows the most abundant viral genera found in each mollusk species according to ecosystem. Taxonomic classification was performed based on Plus PFP database (release 1–12−2024) provided by Kraken2

We identified several predominant phyla of viruses in *D. laeve* and all other mollusks, including Uroviricota (52%), Nucleocytoviricota (15%), Artverviricota (10%), Peploviricota (9%), Pisuviricota (2%), Negarnaviricota (3%), and Preplasmaviricota (1%) (Fig. 3A). These taxa constitute the core viral microbiota as they are consistently among the most abundant viruses across all species of all three ecosystems. One exception to this was *C. gigas* in which Peploviricota predominate. In most mollusk species, the group “Others”, which includes those phyla with less than 1% of relative abundance, was proportionally small.

The virus genus core, the top 10 most abundant across all mollusk species, includes *Alexandravirus* (2%), *Alphabaculovirus* (3%), *Andhravirus* (2%), *Mimivirus* (3%), *Noxifervirus* (1%), *Pandoravirus* (3%), and *Valbvirus* (1%) (Fig. 3B). The full set of the genera detected per species is provided in Supplementary File 2 – Table S3. However, more than half of the total relative abundance was of genera with less than 1% of relative abundance, which were grouped into “Others”. Some genera show higher relative abundance in *D. laeve*, *A. ater*, *B. glabrata*, *P. canaliculata*, and *C. gigas* (Supplemental File 2 - Table S3). This highlights the genus *Metaavulavirus* in the slug *D. laeve*; *Machinavirus*, *Haartmanvirus*, *Erwinia phage*, and *Certrevirus* in the slug *A. ater*; *Orpheovirus* in freshwater snails (*B. glabrata* and *P. canaliculata*); and the particular case of *Ostreavirus* in the oyster *C. gigas*. Moreover, all the seawater mollusks have in common the presence of the genera *Pandoravirus*, *Andhravirus*, and *Valbvirus*.

At the genus level in Viruses and in the other biological domains in the following sections, “NA” groups those sequences that could not be assigned any taxonomical identity.

#### Archaea

**Fig. 4:**
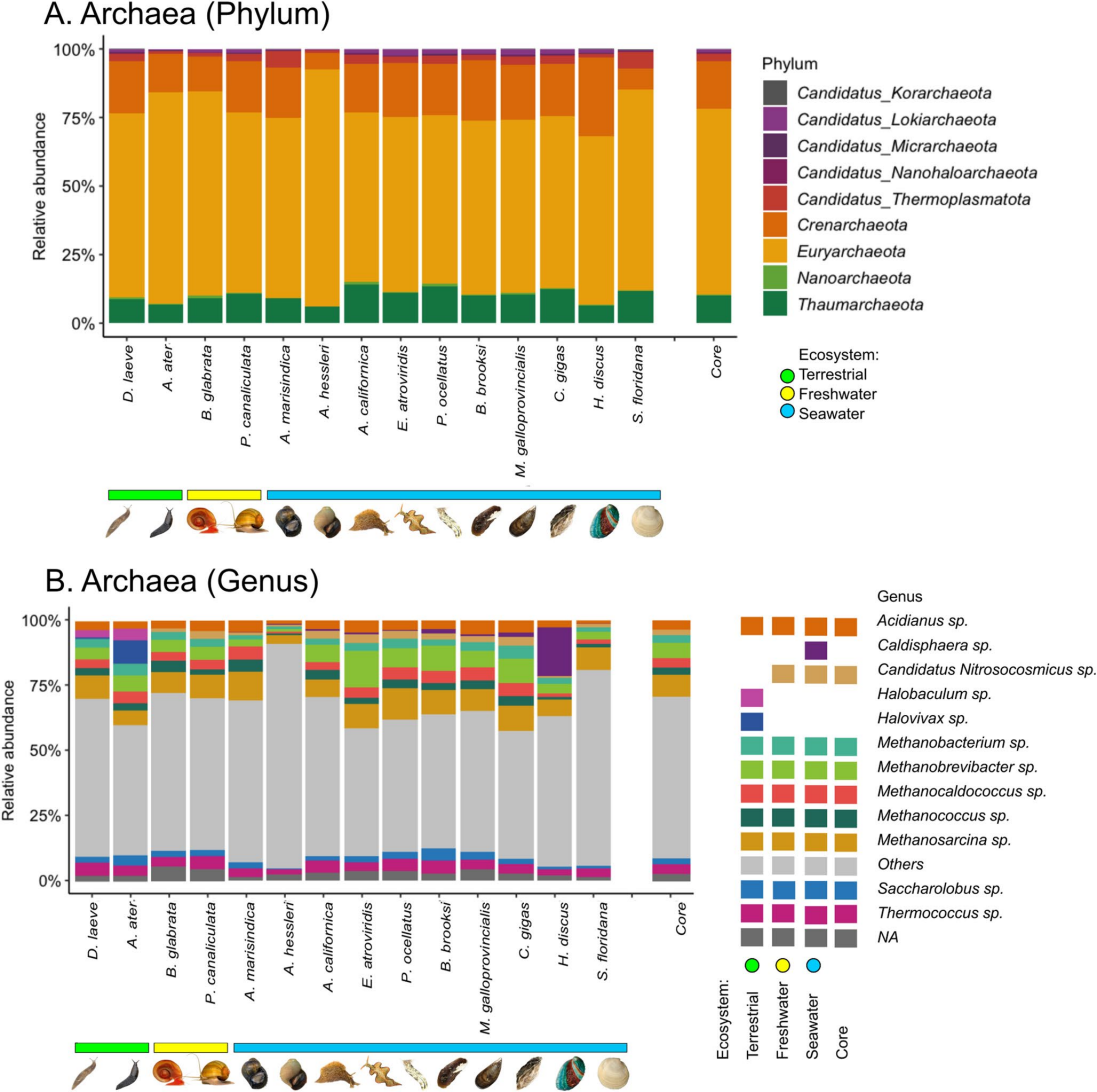
Comparison of the relative abundance of Archaea in the microbiome of *Deroceras laeve* and other mollusks analyzed by shotgun metagenome sequencing. Mollusk species are sorted by the ecosystem occupied by each along the X-axis: terrestrial (green horizontal bar), freshwater (yellow horizontal bar), and seawater (blue horizontal bar). (**A**) Barplot of the top nine phyla and “Others” based on relative abundance. (**B**) Barplot of relative abundance of twelve predominant genera, “Others”, and “NA” (not assigned). On both, A and B, the last barplot shows the core composition of Archaea. The legend shows the most abundant Archaea genera found in each mollusk species according to ecosystem. Taxonomic classification was performed based on Plus PFP database (release 1–12−2024) provided by Kraken2

All mollusk species exhibit a relatively homogeneous phylogenetic diversity in the archaeal domain. The phylum level is mainly shaped by Euryarchaeota (67%), Crenarchaeota (17%), and Thaumarchaeota (10%) (Fig. 4A). This includes diverse groups such as methanogens, halophiles, thermophiles, and acidophiles. Other phyla such as Nanoarchaeota (0.3%), Candidatus Thermoplasmatota (2%), Candidatus Nanohaloarchaeota (0.1%), Candidatus Micrarchaeota (0.2%), Candidatus Lokiarchaeota (1%), and Candidatus Korarchaeota (0.1%) are present, but at lower abundance.

Regarding genera, *Acidianus* (3%), *Candidatus Nitrosocosmicus* (2%), *Methanobacterium* (3%), *Methanobrevibacter* (5%), *Methanocaldococcus* (3%), *Methanococcus* (3%), *Methanosarcina* (7%), *Saccharolobus* (2%), and *Thermococcus* (3%) are ubiquitously present as part of the core (Fig. 4B). Distinctly, the terrestrial slugs (*D. laeve* and *A. ater*) harbor *Halobaculum*, and *Halovivax* was more abundant in *A. ater* (Supplemental File 2-Table S6). Conversely, *Candidatus Nitrosocosmicus* is only present in freshwater and seawater mollusks, while *Caldisphaera* is at high abundance in the abalone *Haliotis discus*. It is noteworthy that the group “Others” similarly defined as in the virus domain, in most mollusk species was negligible at the Archaea phylum level, but it represented more than half the taxonomic assignations at the genus level.

#### Bacteria

**Fig. 5:**
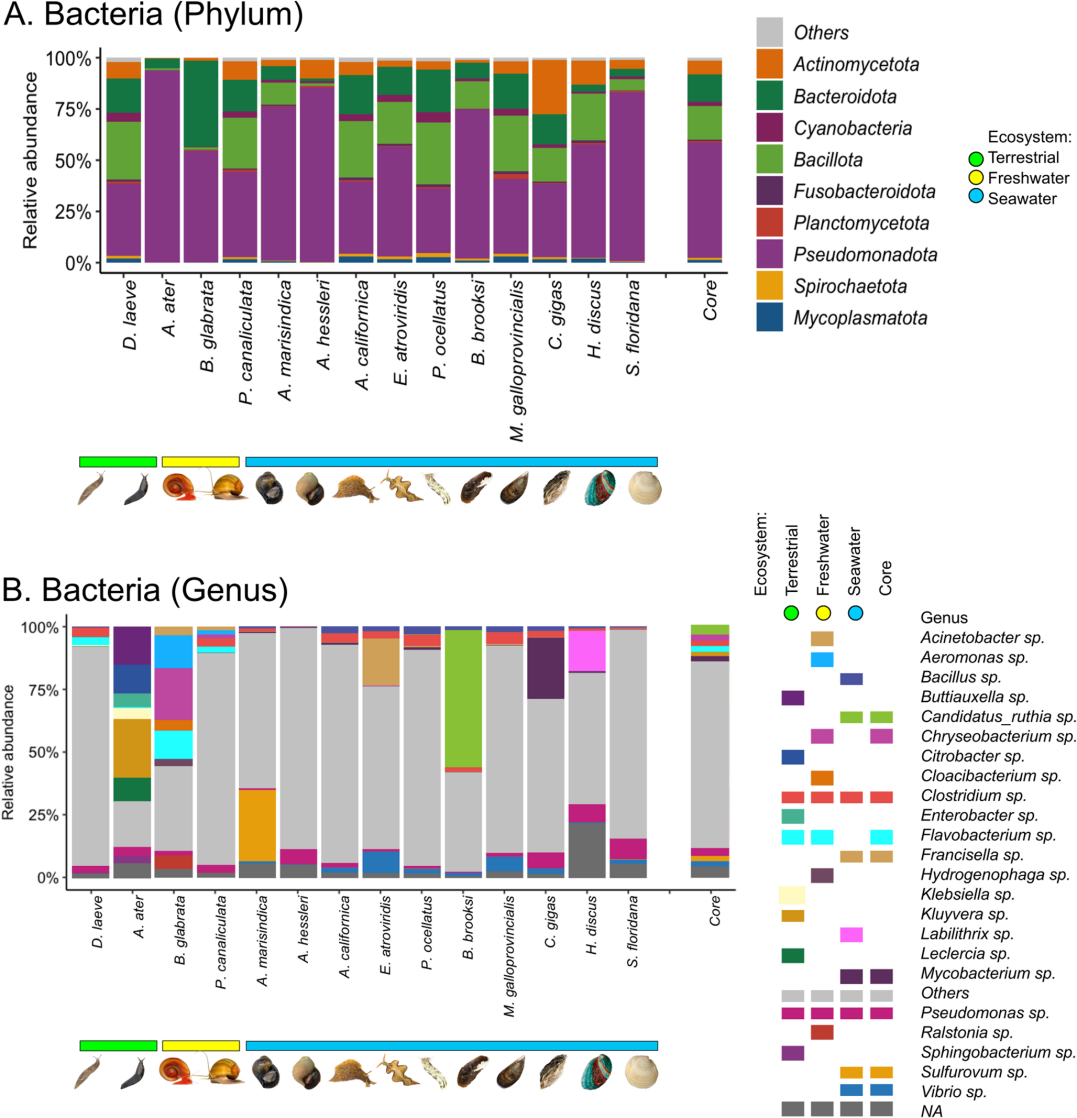
Comparison of the relative abundance Bacteria in the microbiome of *Deroceras laeve* and other mollusks analyzed by shotgun metagenome sequencing. Mollusk species are sorted by the ecosystem occupied by each along the X-axis: terrestrial (green horizontal bar), freshwater (yellow horizontal bar), and seawater (blue horizontal bar). (**A**) Barplot of the top nine phyla and “Others” based on relative abundance. (**B**) Barplot of relative abundance of 23 predominant genera, “Others”, and “NA” (not assigned). On both A and B, the last barplot shows the core composition of Bacteria. The legend shows the most abundant genera of Bacteria found in each mollusk species according to ecosystem. Taxonomic classification was performed based on Plus PFP database (release 1–12−2024) provided by Kraken2

The dominant core of bacterial phyla in the mollusk species contains Pseudomonadota (56%), Bacillota (16%), Bacteroidota (13%), and Actinomycetota (6%), with some fluctuations between species (Fig. 5A). *D. laeve* shares the same structure of the phylum core, whereas *A. ater* is predominantly composed of Pseudomonadota (90%). Regarding freshwater mollusks, *P. canaliculata* is also similar to the phylum core, whereas in *B. glabrata* more than half the abundance corresponds to Pseudomonadota and slightly less than half to Bacteroidota. Marine mollusks (*H. discus hannai*, *A. marisindica*, *M. galloprovincialis*, *B. brooksi*, *C. gigas*, *S. floridana*, *A. hessleri*, *A. californica*, *E. atroviridis*, and *P. ocellatus*) exhibit greater heterogenity with the dominance of Pseudomonadota and appearances of Bacillota, Bacteroidota and Actinomycetota.

At the genus level, the core is composed of *Candidatus Ruthia* (4%), *Chryseobacterium* (2%), *Clostridium* (2%), *Flavobacterium* (2%), *Francisella* (2%), *Mycobacterium* (2%), *Pseudomonas* (2%), *Sulfurovum* (2%), and *Vibrio* (2%) (Fig. 5B). More precisely, *Clostridium* and *Pseudomonas* are distributed across all mollusks, regardless of species. However, the terrestrial gastropods (*D. laeve* and *A. ater*) exhibit notable differences in bacterial composition. *D. laeve* is characterized by the presence of *Clostridium* and *Flavobacterium* and *A. ater* harbors a more diverse set of bacterial genera, including *Citrobacter*, *Buttiauxella*, *Enterobacter*, *Kluyvera*, *Klebsiella*, *Leclercia*, and *Sphingobacterium* (Supplemental File 2 - Table S8), most of which have been previously reported in other mollusks. For freshwater gastropods, *B. glabrata* displays a higher diversity than *P. canaliculata*, including *Acinetobacter*, *Aeromonas*, *Chryseobacterium*, *Cloacibacterium*, *Flavobacterium*, *Hydrogenophaga*, and *Ralstonia*. Seawater mollusks also show more heterogenity and certain genera are species-specific, including *Sulfurovum* in the snail *A. marisindica*, *Francisella* in the slug *E. atroviridis*, *Candidatus Ruthia* in the mussel *B. brooksi*, *Buttiauxella* in the oyster *C. gigas*, and *Labilithrix* in the abalone *H. discus*. These patterns highlight that bacterial diversity varies across mollusk species that occupy different habitats. The group “Others” at the phylum level has a very low abundance in Bacteria in all mollusk species yet at the genus level it represents more than half the abundance in most species.

#### Fungi

**Fig. 6:**
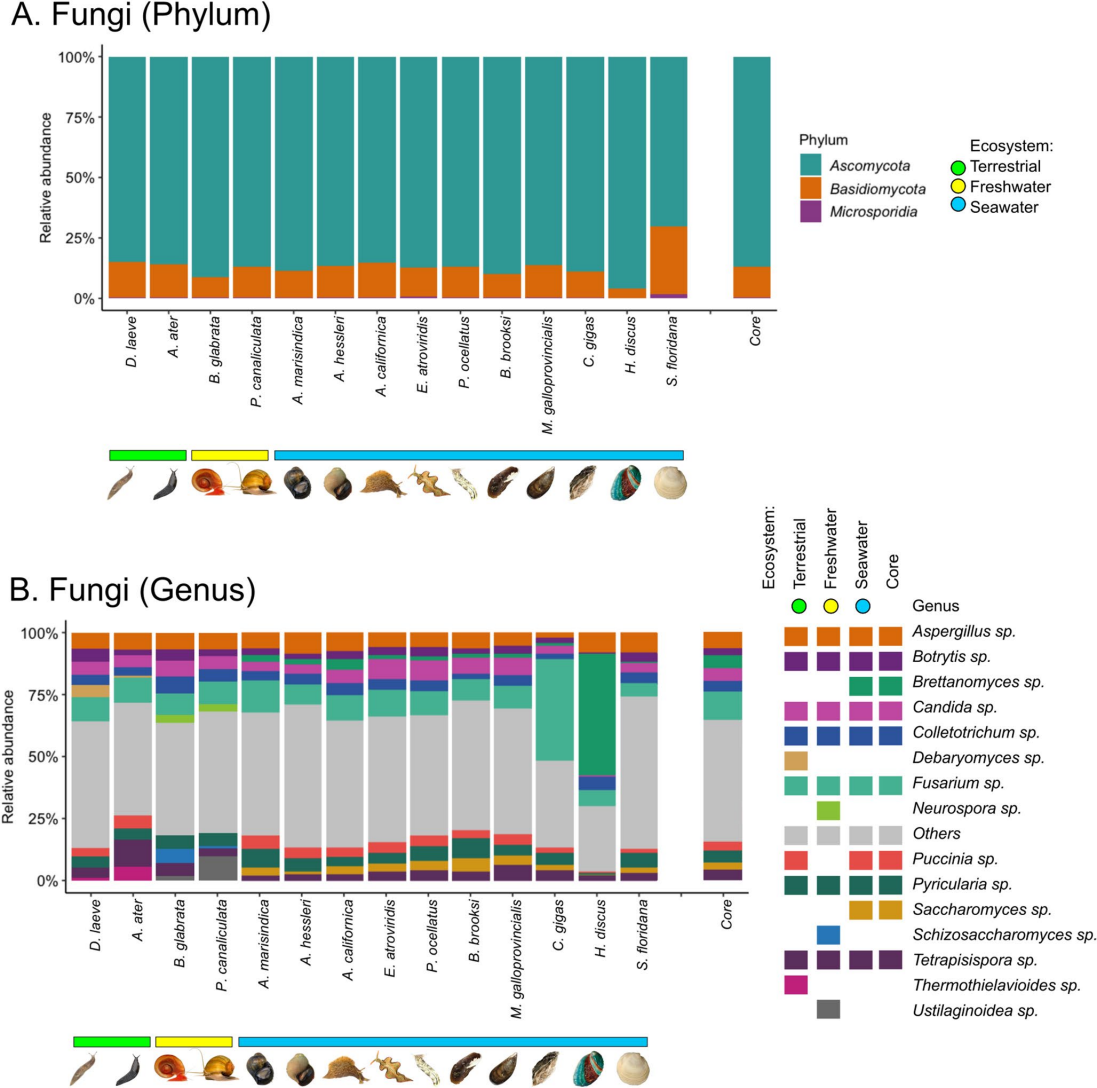
Comparison of the relative abundance Fungi in the microbiome of *Deroceras laeve* and other mollusks analyzed by shotgun metagenome sequencing. Mollusk species are sorted by the ecosystem occupied by each along the X-axis: terrestrial (green horizontal bar), freshwater (yellow horizontal bar), and seawater (blue horizontal bar). (**A**) Barplot of the top three phyla and “Others” based on relative abundance. (**B**) Barplot of relative abundance of fourteen predominant genera and “Others”. On both A and B, the last barplot shows the core composition of Fungi. The legend shows the most abundant genera of Fungi found in each mollusk species according to ecosystem. Taxonomic classification was performed based on Plus PFP database (release 1–12−2024) provided by Kraken2

Ascomycota is the dominant fungal phylum (at approximately 86%) in all mollusks, while Basidiomycota appears at a lower proportion with a relative abundance of about 13% (Fig. 6A). Microsporidia are present with an abundance of less than 1%, with the exception of *S. floridana*, in which it is slightly more than 1%. This fungal composition remains relatively consistent between mollusks, with no significant differences regarding ecosystem, tissue sample, or mollusk type. In fact, the fungal genus core reveals the consistent presence of *Aspergillus* (7%), *Botrytis* (3%), *Brettanomyces* (6%), *Candida* (5%), *Colletotrichum* (4%), *Fusarium* (11%), *Puccinia* (4%), *Pyricularia* (5%), *Saccharomyces* (3%), and *Tetrapisispora* (4%), among all mollusks (Fig. 6B).

At the genus level, we found few differences. Namely, the genera of *Debaryomyces* in the mycobiome of *D. laeve* and *Thermothielavioides* in *A. ater* (terrestrial slugs) are prominent (Supplemental File 2 - Table S10), in contrast to the relevant genus *Neurospora* that is present only in freshwater mollusks (*B. glabrata* and *P. canaliculata*). Among seawater mollusks, *Saccharomyces* and *Brettanomyces* were the most prevalent fungal genera, with *Brettanomyces* particularly abundant in *H. discus*. The group “Others” is scanty in Fungi at the phylum level in all mollusk species and it represents more than half the abundance in most species at the genus level.

### Beta Diversity

**Fig. 7:**
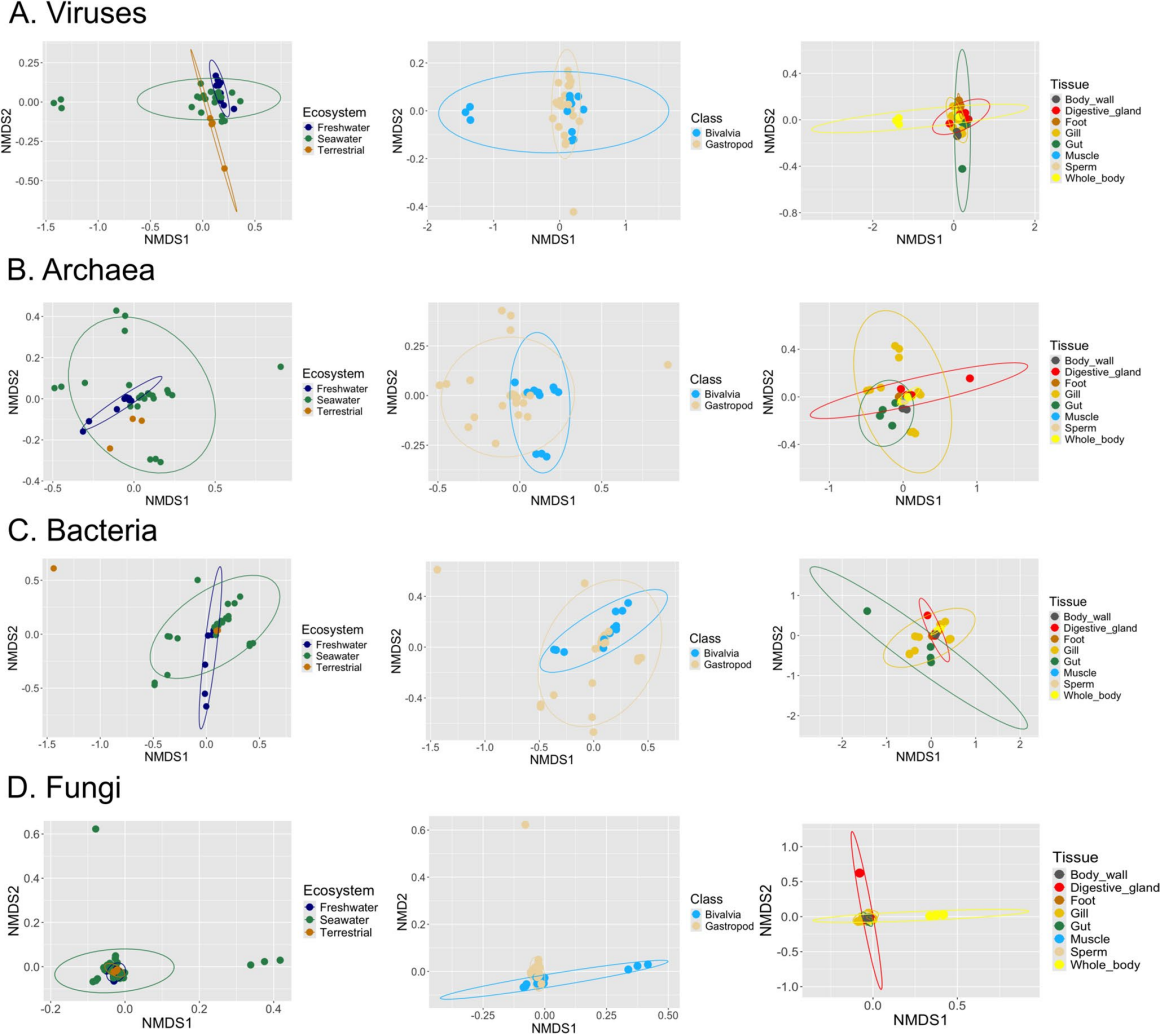
Analysis of similarities between the microbiome of the fourteen mollusk species by Non-metric MultiDimensional Scaling (NMDS). (**A**) Viruses. (**B**) Archaea. (**C**) Bacteria. (**D**) Fungi. Left panels are based on ecosystem: freshwater (blue), seawater (green), and terrestrial (orange brown). Central panels are based on Class of mollusk: bivalvia (light blue) and gastropod (tan). Right panels are based on sampled tissue: body wall (dark grey), digestive gland (red), foot (orange brown), gill (gold), gut (green), muscle (light blue), sperm (tan), and whole body (yellow)

To better understand the structure of molluscan microbiomes, we performed an analysis that categorizes similarities of mollusk species in a lower-dimensional space based on their microbial composition (NMDS). The visualization by ecosystems (Fig. 7) indicates that viral and fungal communities clustered more tightly, whereas archaeal and bacterial communities exhibited greater variation across samples. The beta-diversity results suggest a stronger relationship in viral and fungal communities in gastropod species (*A. ater*, *B. glabrata*, *P. canaliculata*, *H. discus hannai*, *A. marisindica*, *A. hessleri*, *A. californica*, *E. atroviridis*, and *P. ocellatus*), while archaea and bacteria are more related for bivalves (*M. galloprovinciallis*, *B. brooksi*, *C. gigas*, and *S. floridana*). The PERMANOVA analyses (Supplemental File 1 – Table S11-S15) revealed that multiple factors significantly influence the composition of the mollusk microbiome, with species, tissue type, and ecosystem showing particularly strong effects. Moreover, pairwise comparison revealed that among tissues, the body wall and gut had the highest impact, especially on fungal (R² = 0.56) and bacterial (R² = 0.48) communities, respectively, while the foot tissue also showed marked differentiation across microbial groups, including archaea and viruses. Regarding ecosystems, freshwater environments exerted the strongest influence on structure of the microbiota across all domains, with the most pronounced differences observed in viral (R² = 0.22) and fungal (R² = 0.21) communities.

### Functional Profiling of Mollusk Microbiomes

**Fig. 8:**
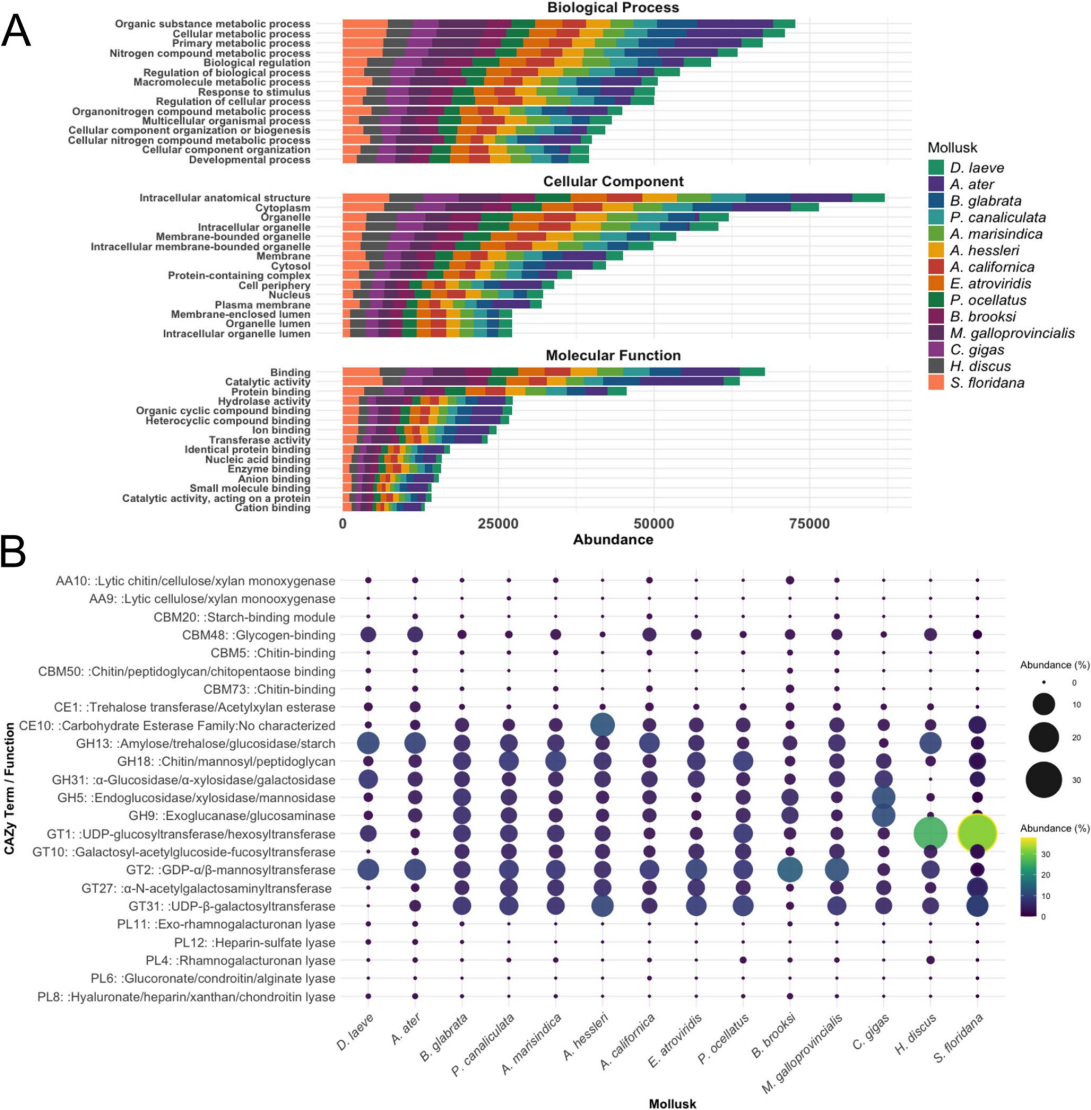
Functional fingerprinting of the microbiome of the fourteen mollusk species based on eggNOG annotation with the v2.1.9 database. (**A**) Barplots of top fifteen Gen Ontology (GO) terms for the three categories: Biological Process, Cellular Component, and Molecular Function. (**B**) Bubble plot of the top five most abundant Carbohydrate-Active enzyme families (CAZy). PL: Polysaccharide Lyase Family, GT: Glycosyl Transferase Family, GH: Glycoside Hydrolase Family, CE: Carbohydrate Esterase Family, CBM: Carbohydrate-Binding Module Family, AA: Auxiliary Activity Family

To assess potential roles in the interaction of the microbiome with the host, we analysed the functional annotation of the predicted proteins of the microbiomes from the mollusk species in this study (eggNOG functional categories, CAZy enzyme families, and KEGG metabolic pathways). In general, all mollusk species present a similar pattern in gene ontology abundance (Fig. 8A).

The most abundant biological processes are metabolism of organic substances (9.2%), cellular processes (9.0%), primary processes (8.5%), nitrogen compound metabolism (8.0%), macromolecule metabolism (6.4%), organo-nitrogen compound metabolism (5.9%), and cellular nitrogen compound metabolism (5.0%). The most abundant categories in cellular components are intracellular anatomical structures (12.5%), cytoplasm (11.%), organelle components (8.9%), intracellular components (8.7%), extracellular membrane-bound components (7.7%), and intracellular membrane-bound components (7.1%), with the least abundant being those associated with organelle lumen (3%). Regarding molecular functions, the most abundant are binding (16.4%), catalytic activity (15.4%), protein binding (11.06%), hydrolase activity (6.6%), and binding to various compounds such as organic cyclic compounds (6.5%), heterocyclic compounds (6.4%), ions (5.9%), enzymes (3.8%), and cations (3.1%). We also analyzed the enrichment of carbohydrate-active enzyme families (Fig. 8B). The main enzyme encoding-genes include the glycosyltransferases GT1, GT2, GT10, GT27 and GT31 (57.1%); followed by the glycoside hydrolases GH5, GH9, GH13, GH18 and GH31 (32.5%); the carbohydrate esterases CE1 and CE10 (7.0%); then the carbohydrate-binding module proteins CBM5, CBM20, CBM48, CBM48, CBM50, and CBM73 (2.8%); and at a lower abundance, the polysaccharide lyases PL4, PL6, PL8, PL11 and PL12 (0.3%) and the auxiliary activity enzymes AA9 and AA10 (0.2%). The most abundant Kyoto Encyclopedia of Genes and Genomes (KEGG) Orthology (KO) pathways were also identified for the mollusk species of this study. The top 30 Level B pathways were consolidated into seven Level A categories: Metabolism, Genetic information processing, Environmental information processing, Cellular processes, Organismal systems, Human diseases, and Brite hierarchies (Supplementary File 1 – Figure S4).

## Discussion

The microbiome of every organism is related to environmental adaptability, metabolic regulation and functional profiling [1]. Despite the high relevance and abundance of mollusks in the animal domain, few studies have been conducted to provide insight into their symbiotic organisms. Here we show that the few mollusk species for which shotgun metagenomic data exists belong to the three divergent branches of heterobranchs, caenogastropod/vetigastropod, and bivalves. Despite their various environments, however, their microbial communities have a common core, although important differences were also observed.

The rarefaction curves reveal a complex microbiota with good coverage of taxonomical identification, similar to reports from other mollusks such as *B. straminea*, *B. aeruginosa*, *P. acuta*, *P. corneus*, and *P. canaliculata* [19]. This comparison revealed similar general patterns which should nevertheless be interpreted with caution owing to methodological differences in sequencing approaches [20]. In our study, alpha diversity varied significantly across species, ecosystems, and mollusk types. The influence of environmental factors such as seasonal variations, wild habitats, and even the dietary patterns on microbial diversity of mollusks have been documented in previous reports. The Shannon index of the fecal bacteriome of wild *Ariolimax columbianus* (pacific banana slug) is significantly lower during spring (1.74) than during fall (2.12), owing to feeding on ephemeral spring plants and fall leaf litter, respectively [10]. Likewise, the Shannon index of the whole body bacteriome of the slug *Ambigolimax valentianus* is significantly lower under an unsterile diet than on a sterile diet, associated with changes in nutrients or resident microbiota induced by the sterilization process [21]. In our study, pairwise PERMANOVA comparisions revealed body wall, gut, and freshwater ecosystems as the most influential factors shaping mollusk-associated microbial communities.

Reports of viruses in terrestrial slugs are scarce. Here, Uroviricota was the most abundant phylum in *D. laeve*. It comprises double-stranded DNA-tailed bacteriophages of the *Erwinia phage* group that usually infect plant pathogen bacteria [22], and *Certrevirus* which is associated with black-leg in potato fields [23]. In *D. laeve* we identified the genus *Alphabaculovirus*, known to infect insect larvae such as the zebra caterpillar [24], and *Metaavulavirus* which infects a wide spectrum of birds [25], neither of which have been reported previously in mollusks. Another relevant finding was the high abundance of *Ostreavirus* in *C. gigas* which is linked to a disease outbreak in oyster farms in Brittany, France [26]. In addition, the core is shaped by *Pandoravirus*, reported in freshwater ponds [27], but not previously observed in mollusks. These findings suggest terrestrial and other mollusks could act as reservoirs of viruses and carry them through gardens, lands, freshwater, and seawater environments.

The archaeome structure associated with mollusks is also poorly documented. Regarding Archaea, the predominance of Euryarchaeota and Crenarchaeota, which usually thrive in extreme environments, as well as Thaumarchaeota suggests their symbiosis with mollusks. The Euryarchaeota include the genera *Acidianus*, *Methanobacterium*, *Methanobrevibacter*, *Methanocaldococcus*, *Methanococcus*, *Methanosarcina*, *Thermococcus*, and *Saccharolobus*, suggesting a syntrophic interaction related to carbon, as this phylum has been associated with methanogenesis [28] and sulfur cycles [29]. We also detected the presence of the halophilic *Halobaculum* for the first time in terrestrial slugs (*D. laeve* and *A. ater*), contrasting with their usual presence in hypersaline lakes, soda lakes, solar salterns, and salt mines [30]. Another relevant phylum is Crenarchaeota that includes *Candidatus Nitrosocosmicus*, which we found in freshwater and seawater mollusks and has been associated with the ammonia oxidation process [31]. Our finding of the genus *Caldisphaera* in the abalone *Haliotis discus* also contrasts with its isolation from hot springs or geothermal environments and also suggests a specific interaction [32].

The bacteriome is the most reported portion of the mollusk microbiome, although it has been thus far only documented partially. Similarly to other work, the phylum Pseudomonadota predominates in terrestrial slugs such as *A. ater* [33], *A. columbianus* [10], and the filter-feeding clam *M. edulis* [34]. At the genus level a larger diversity was observed. *D. laeve* has *Clostridium and Flavobacterium* which have also been found as a constituent of gut microbiome of freshwater mussels (*C. asperata*, *F. cerina*, *L. ornata*, and *O. unicolor*) [35]. *A. ater* holds *Citrobacter* and *Enterobacter*, also documented previously in the same species [33] and in the snail *H. pomatia* [36]. The genera *Klebsiella* and *Buttiauxella* were also found in *A. ater* in our study. The former has been reported in abalone [37], marine bivalves [38], and gastropods [39], while the latter in the slug *Geomalacus maculosus* [40] and snail *Cornu aspersum* [41]. In the case of *Sphingobacterium* found in *A. ater* herein, it was also reported in the snail *Achatina fulica* [8].

These findings suggests a symbiotic association between mollusks and gamma-pseudomonadota. An example of the type of potential interactions with their host is illustrated by the presence in *A. ater* of *Kluyvera*, shown to have endoglucanase and beta-glucosidase activity [42]. This association may be functionally relevant in the degradation of plant-derived polysaccharides, a role commonly attributed to microbial symbionts in invertebrate digestive systems. An unexpected finding in freshwater mollusks, however, was *Aeromonas*, a member of gamma-pseudomonadota previously reported mostly in terrestrial mollusks and linked to cellulose degradation pathways [42]. Its detection in freshwater mollusks is therefore considered particularly unusual. Furthermore, in line with Newton et al. [43] we suggest that *Candidatus Ruthia* could be the most abundant chemoautotrophic endosymbiont in the seawater *B. brooksi*. Finally, the core bacteriome identified in this work is consistent with reports on *Elysia chlorotica* [44], *Pacific oyster* [45], *Ariolimax columbianus* [10], *A. ater* [42], and *B. glabrata* [6].

The mollusk mycobiome is the second most documented after the bacteriome, although there is still much to discover. The high abundance of *Debaryomices* and *Thermothielavioides* in *D. laeve* and *A. ater* suggests a symbiotic relationship. These nonpathogenic yeast indicate a fungal community shaped by detritivorous feeding behavior in soil-rich environments with high salinity, where organic matter and plant debris are abundant. In fact, these genera of fungi are used industrially for the degradation of biomass into biofuel [46] and also as fermentors in food [47] and probiotics for animals [48]. Similarly, the abundance of *Neurospora* suggests symbiosis in the case of freshwater species in a habitat driven by vegetation or sediments of organic matter. These fungi are typically found in decaying organic matter and are also used in food production [49]. Other relevant genera are *Aspergillus* [50], *Botrytis* [51], and *Fusarium* [52] which comprise the fungal core and have also been reported in mussels and bivalves. Unexpected was the presence of *Brettanomyces* and *Saccharomyces* in seawater species, since they have not been detected in marine mollusks. These yeast-like species could be related to dietary intake of organic detritus in coastal zones. In association with the diversity analysis, these findings suggest a degree of dietary specialization among fungi and their mollusk hosts.

The functional annotation analysis of the mollusk metagenomes supports the interpretation that the protein machinery of the entire microbiome, along with the presence of specific phyla such as Gamma-Pseudomonadota and Ascomycota, may contribute to the metabolism of various compounds. This is evidenced by the enrichment of key biological processes and molecular functions, including catalytic, hydrolase, and compound-binding activities. Similarly, the enrichment of the enzymatic machinery confirms the presence of glycosyltransferases, glycoside hydrolases, carbohydrate esterases, and polysaccharide lyases involved in the fermentation and metabolism of both simple and complex carbohydrates. In addition, the presence of methane metabolism pathways in all mollusk samples may be linked to methanogenic archaea. Additionally, among the most prominent pathways identified across all mollusks is the mitogen-activated protein kinase (MAPK) pathway associated with yeasts, which has been shown to respond to a variety of stimuli including pheromone signals, osmotic stress, cell wall perturbations, and nutritional status [53]. Other relevant pathways include lipid biosynthesis, vesicular membrane transport, and cell signaling mediated by exosomes. The latter underscores the significance of host–microbiome interactions, as exosomes may modulate homeostasis of microbiota in the hemolymph as has been reported during pathogenic infections in the crustacean *Scylla paramamosain* [54].

Recent findings in cephalopods have revealed that microbial communities participate in shaping the sensory world of their animal hosts [55]. It is therefore plausible that mollusk microbiomes, especially in species with developed sensory systems or behavioral plasticity, may also contribute to environmental perception, suggesting a deeper level of functional integration between microbiomes and host biology than previously assumed. Notably, the overall structural consistency of microbial communities across mollusk species suggests a long-term coevolutionary relationship, reinforcing the hypothesis that microbe–host interactions have played a role in shaping dietary adaptations in terrestrial gastropods, analogous to patterns documented in insects [56].

## Conclusions

This study represents the first comprehensive analysis of the microbiome of *D. laeve* and the core microbiome of various mollusk species. The body wall of *D. laeve* slugs is colonized by a complex microbial community that is expected to be stable as the animals studied were kept under laboratory conditions for a year. The main viruses are phages of plant pathogens and other bacteriome interactors. The archaea community includes mainly halophiles and methane-processing organisms. The bacterial community is made up mainly of producers of cellulose hydrolases, and the fungal community mainly of fermenters and producers of lignin or chitin extracellular enzymes. An additional relevant finding was that for each domain, a single phylum was predominant across all species and tissues studied, despite marked differences at the genus level and their beta diversity clustering by environments. Hence, Uroviricota, Euryarchaeota, Pseudomonadota, and Ascomycota are dominant phyla across mollusk species with the notable exception of Peploviricota among viruses in *C. gigas*, which has been linked to an infection by *Ostreavirus sp*. This highlights a substantial opportunity for the discovery of previously unknown host–microbe interactions through specifically-designed sampling protocols aimed to overcome the limitations of the present study, namely, the heterogeneity in samples derived from independent studies. Nevertheless, the microbiome characterized in *D. laeve* lays the groundwork for future studies on host–symbiont interactions. Understanding these relationships may shed light on physiological processes and ecological advantages that contribute to the remarkable adaptability and global distribution of this species. Importantly, our findings open avenues for targeted microbiome manipulation and experimental studies to decipher or enhance symbiotic functions, with potential implications in ecology, conservation, and biotechnology.

## Supplementary Information

Below is the link to the electronic supplementary material.

Supplementary Material 1 (XLSX 6.04 MB)

Supplementary Material 2 (DOCX 16.2 MB)

## Data Availability

The raw sequences data have the accession number PRJNA1033643 BioProject of Sequence Read Archive (SRA) database from NCBI.
