# Supplementary material for: The Body Wall Microbiome of the Terrestrial Slug Deroceras laeve Reveals Potential Endosymbionts and Shares Core Organisms with Other Mollusks
Source: Microb Ecol. 2025 Nov 18;88:136. doi: 10.1007/s00248-025-02652-8 (PMC12664854; doi:10.1007/s00248-025-02652-8)
Supplement: Supplementary file 1 [file 248_2025_2652_MOESM2_ESM.docx]

**Fig. S1**. Phylogenetic tree of the thirteen mollusk species analysed in this study based on the sequences of Cytochrome oxidase 1 (COX1). The numbers at the nodes indicate the confidence support values by 1000 bootstrap replicates. The blue shadow shows bivalves and the orange shadow shows gastropods. Color code of habitats: green, terrestrial; yellow, freshwater; and blue, seawater. The COX1 gene sequence is unknown for *Stewartia floridana*.

**Fig. S2**. Global taxonomy of the microbiome of the body wall of *Deroceras laeve*. The Sankey diagram shown was performed using the Kraken2 taxonomy report denoting domain (D), phylum (P), Class (C), Order (O), Family (F) and Genus (G). The thickness of each flow arrow represents the quantity of classified reads. The numerical value above each node indicates the count of k-mer hits.

**Fig. S3**. Taxonomy assignment of the mollusk microbiomes. Rarefaction curves obtained by *rrarefy* are shown for A) Viruses, B) Archaea, C) Bacteria, and D) Fungi. The X-axis shows the number of sequences, and the Y-axis shows the number of identified species (IDs). The color code of the line for each species is shown at the bottom of the figure.

**Fig. S4.** Heatmap of Kyoto Encyclopedia of Genes and Genomes (KEGG) orthology (KO) pathways. The rows show the top 30 KEGG level-C / level-B pathways and order by KEGG level-A categories. The columns shows the mean of the pathway category for each mollusk specie.

**Table S1.** General features including the code of Sequence Read Archive (SRA) from National Center for Biotechnology Information (NCBI), sample names, number of reads, origin of tissue sample and sequencing platform for each shotgun metagenomic dataset of the fourteen mollusk species. The mollusks species are ordered by ecosystem: terrestrial, freshwater, and seawater. The sample column lists the names assigned in the figures.

| **Mollusk specie** | **SRA** | **Sample** | **Raw reads**  **(M)** | **Quality reads (M)** | **Tissue** | **Sequencing platform** | **Reference** |
| --- | --- | --- | --- | --- | --- | --- | --- |
| **Terrestrial** |  |  |  |  |  |  |  |
| *Deroceras laeve* | BC59 (SRR32066467) | D. laeve | 2,6 | 2,4 | Body wall | Illumina NextSeq 500 | This project |
|  | BC60 (SRR32066466) | D. laeve | 2,0 | 1,8 |  |  |  |
|  | BC61 (SRR32066465) | D. laeve | 2,7 | 2,4 |  |  |  |
|  | BC62 (SRR32066464) | D. laeve | 2,5 | 2,3 |  |  |  |
|  | BC63 (SRR32066463) | D. laeve | 2,4 | 2,1 |  |  |  |
| *Arion ater* | ERR2021710 | A. ater | 12,9 | 9,9 | Gut | Illumina MiSeq | [33] |
| **Freshwater** |  |  |  |  |  |  |  |
| *Biomphalaria glabrata* | SRR16036425 | B. glabrata | 16,9 | 13,7 | Gut | Illumina HiSeq 2500 | [6] |
|  | SRR16036427 | B. glabrata | 17,9 | 14,9 |  |  |  |
|  | SRR16036451 | B. glabrata | 17,0 | 14,1 |  |  |  |
| *Pomacea canaliculata* | SRR24819716 | P. canaliculata | 21,7 | 15,7 | Foot | Illumina NovaSeq 6000 | [57] |
|  | SRR24819717 | P. canaliculata | 21,8 | 14,9 |  |  |  |
|  | SRR24819719 | P. canaliculata | 21,7 | 15,7 |  |  |  |
|  | SRR24819751 | P. canaliculata | 20,2 | 13,9 |  |  |  |
|  | SRR24819754 | P. canaliculata | 20,3 | 14,0 |  |  |  |
|  | SRR24819759 | P. canaliculata | 21,3 | 14,6 |  |  |  |
| **Seawater** |  |  |  |  |  |  |  |
| *Haliotis discus hannai* | SRR2912835 | H. discus | 5,8 | 3,8 | Digestive gland | Illumina MiSeq | Unpublished |
| *Alviniconcha marisindica* | SRR11781617 | A. marisindica | 19,9 | 13,7 | Gill | Illumina NovaSeq 6000 | [58] |
|  | SRR11781621 | A. marisindica | 20,2 | 13,9 |  |  |  |
|  | SRR24819719 | A. marisindica | 19,8 | 13,9 |  |  |  |
| *Mytilus galloprovincialis* | ERR11641685 | M. galloprovincialis | 6,2 | 4,4 | Digestive gland | Illumina NextSeq 550 | [59] |
|  | ERR11641686 | M. galloprovincialis | 10,8 | 7,6 |  |  |  |
|  | ERR11641687 | M. galloprovincialis | 15,2 | 10,9 |  |  |  |
| *Bathymodiolus brooksi* | SRR8426922 | B. grooksi | 37,4 | 27,9 | Gill gland | Illumina HiSeq 2500 | [60] |
|  | SRR8426926 | B. grooksi | 37,2 | 26,7 |  |  |  |
|  | SRR8426928 | B. grooksi | 26,0 | 10,9 |  |  |  |
| *Crassostrea gigas* | SRR13169036 | C. gigas | 59,7 | 37,4 | Whole body | Illumina NovaSeq 6000 | [25] |
|  | SRR13169040 | C. gigas | 53,8 | 33,8 |  |  |  |
|  | SRR13169045 | C. gigas | 60,8 | 38,6 |  |  |  |
| *Stewartia floridana* | SRR5873458 | S. floridana | 11,1 | 7,1 | Gill | Illumina HiSeq 2500 | [61] |
|  | SRR5873459 | S. floridana | 8,6 | 5,6 |  |  |  |
|  | SRR5873460 | S. floridana | 7,8 | 4,5 |  |  |  |
| *Alviniconcha hessleri* | SRR15909356 | A. hessleri | 26,2 | 22,2 | Gill | Illumina MiSeq | [62] |
|  | SRR15909357 | A. hessleri | 24,0 | 20,7 |  |  |  |
|  | SRR15909359 | A. hessleri | 30,3 | 26,7 |  |  |  |
| *Aplysia californica* | SRR9945487 | A. californica | 349,2 | 233,4 | Whole body | Illumina HiSeq 4000 | [63] |
|  | SRR402292 | A. californica | 51,0 | 28,4 |  |  |  |
| *Elysia atroviridis* | SRR20012986 | E. atroviridis | 27,4 | 20,3 | Muscle gland | Illumina MiSeq | Unpublished |
| *Plakobranchus ocellatus* | SRR11241676 | P. ocellatus | 111,7 | 79,7 | Whole body | Illumina MiSeq | Unpublished |

**Table S2.** General features including Class, Superorder/Superfamily, type of mollusk and ecosystem information of the fourteen mollusk species analyzed. The SRA-COX column lists the NCBI code of cytochrome oxidase 1 (COX1) from each mollusk species.

| **Mollusk** | **Class** | **Superorder*/*Superfamily** | **Type** | **Ecosystem** | **SRA-COX** |
| --- | --- | --- | --- | --- | --- |
| **Terrestrial** |  |  |  |  |  |
| *Deroceras laeve* | Gastropod | Stylommatophora*/*Heterobranchia | Slug | Terrestrial | NC 072953.1 |
| *Arion ater* | Gastropod | Stylommatophora | Slug | Terrestrial | MW659465.1 |
| **Freshwater** |  |  |  |  |  |
| *Biomphalaria glabrata* | Gastropod | Hygrophila*/*Heterobranchia | Snail | Freshwater | NC 005439.1 |
| *Pomacea canaliculata* | Gastropod | Mesograstropoda | Snail | Freshwater | NC 024586.1 |
| **Seawater** |  |  |  |  |  |
| *Haliotis discus hannai* | Gastropod | Lepetellida | Abalone | Seawater | NC 031362.1 |
| *Alviniconcha marisindica* | Gastropod | Caenogastropoda | Snail | Seawater | NC 079669.1 |
| *Mytilus galloprovincialis* | Bivalvia | Mytiloida | Mussel | Seawater | NC 006886.2 |
| *Bathymodiolus brooksi* | Bivalvia | Mytiloida | Mussel | Seawater | NC 059706.1 |
| *Crassostrea gigas* | Bivalvia | Ostreoida | Oyster | Seawater | NC 001276.1 |
| *Stewartia floridana* | Bivalvia | Lucinida | Clam | Seawater | Unknown |
| *Alviniconcha hessleri* | Gastropod | Caenogastropod | Snail | Seawater | MZ823268.1 |
| *Aplysia californica* | Gastropod | Sacoglossa*/*Opisthobranchia | Slug | Seawater | NC 005827.1 |
| *Elysia atroviridis* | Gastropod | Sacoglossa*/*Opisthobranchia | Slug | Seawater | NC 064504.1 |
| *Plakobranchus ocellatus* | Gastropod | Sacoglossa*/*Opisthobranchia | Slug | Seawater | AB501307.1 |

**Table S11**. PERMANOVA (Permutational Multivariate Analysis of Variance) analysis of the metagenomic composition of Viruses, Archaea, Bacteria, and Fungi in relation to Class, Species, type of mollusk, ecosystem, and tissue. Df: degrees of freedom; SS: sum of squares; Pseudo-F: F-value derived from permutation 999 permutations testing. Statistical significance is shown by an asterisk.

|  | Df | SS | R2 | F | Pseudo f (>F) |
| --- | --- | --- | --- | --- | --- |
| **Virus** |  |  |  |  |  |
| Main efects |  |  |  |  |  |
| Class | 1 | 1.0015 | 0.07103 | 2.5998 | 0.001 *** |
| Specie | 13 | 10.4728 | 0.74279 | 4.8872 | 0.001 *** |
| Type | 5 | 4.7822 | 0.33918 | 3.0797 | 0.001 *** |
| Ecosystem | 2 | 1.9309 | 0.13695 | 2.6182 | 0.001 *** |
| Tissue | 7 | 5.4354 | 0.38551 | 2.5095 | 0.001 *** |
| **Archaea** |  |  |  |  |  |
| Main efects |  |  |  |  |  |
| Class | 1 | 0.4582 | 0.08263 | 2.9723 | 0.003 ** |
| Specie | 13 | 4.7586 | 0.85805 | 9.7645 | 0.001 *** |
| Type | 5 | 1.8852 | 0.33993 | 2.987 | 0.001 *** |
| Ecosystem | 5 | 1.8852 | 0.33993 | 2.987 | 0.001 *** |
| Tissue | 7 | 1.7924 | 0.32319 | 1.8419 | 0.005 ** |
| **Bacteria** |  |  |  |  |  |
| Main efects |  |  |  |  |  |
| Class | 1 | 0.5277 | 0.06259 | 2.1368 | 0.02 * |
| Specie | 13 | 7.6911 | 0.91238 | 16.02 | 0.001 *** |
| Type | 5 | 2.8815 | 0.34183 | 2.9084 | 0.001 *** |
| Ecosystem | 2 | 0.8890 | 0.10546 | 1.8273 | 0.027 * |
| Tissue | 7 | 3.3782 | 0.40075 | 2.484 | 0.001 *** |
| **Fungi** |  |  |  |  |  |
| Main efects |  |  |  |  |  |
| Class | 1 | 0.23554 | 0.10333 | 3.918 | 0.001 *** |
| Specie | 13 | 2.02414 | 0.88796 | 13.412 | 0.001 *** |
| Type | 5 | 1.40712 | 0.61728 | 9.6773 | 0.001 *** |
| Ecosystem | 2 | 0.19398 | 0.0851 | 1.5347 | 0.08 . |
| Tissue | 7 | 0.77862 | 0.34157 | 2.075 | 0.01 ** |

Signif. Codes: 0 ‘***’ 0.001 ‘**’ 0.01 ‘*’ 0.05 ‘.’ 0.1 ‘ ’ 1.

**Table S12.** Pairwise PERMANOVA comparison of the metagenomic composition of Viruses across ecosystems and tissue. P-values were adjusted for multiple testing using the Benjamini–Hochberg false discovery rate (FDR) correction. Df: degrees of freedom; Sum of Sqs: sum of squares; R²: proportion of variance explained; F: pseudo-F statistic; P value: uncorrected p-value; P adj: FDR-adjusted p-value.

| ***Comparison by ecosystem*** | Df | Sum Of Sqs | R^2^ | F | *P* value | *P* adj |
| --- | --- | --- | --- | --- | --- | --- |
| Freshwater vs Seawater | 1 | 1.00477591 | 0.08723103 | 2.67588935 | 0.001 | 0.003 |
| Freshwater vs Terrestrial | 1 | 0.95714402 | 0.22122489 | 3.12474517 | 0.002 | 0.003 |
| Seawater vs Terrestrial | 1 | 0.95829284 | 0.09644091 | 2.45489300 | 0.004 | 0.004 |
| ***Comparison by tissue*** | Df | Sum Of Sqs | R^2^ | F | *P* value | *P* adj |
| Foot vs Gill | 1 | 0.98799989 | 0.16092902 | 3.06870864 | 0.001 | 0.015 |
| Gill vs Whole body | 1 | 1.11988126 | 0.17804996 | 3.24928439 | 0.002 | 0.015 |
| Body wall vs Gill | 1 | 1.07430538 | 0.20383290 | 3.32823060 | 0.007 | 0.0175 |
| Digestive gland vs Foot | 1 | 0.77984339 | 0.25722140 | 2.77036947 | 0.005 | 0.0175 |
| Foot vs Gut | 1 | 0.82067720 | 0.27194361 | 2.98815979 | 0.007 | 0.0175 |
| Foot vs Whole body | 1 | 1.15022706 | 0.31499382 | 4.13856761 | 0.004 | 0.0175 |
| Digestive gland vs Gill | 1 | 0.78635051 | 0.13779263 | 2.23739315 | 0.013 | 0.024375 |
| Gill vs Gut | 1 | 0.83714449 | 0.14679662 | 2.40874882 | 0.012 | 0.024375 |
| Body wall vs Foot | 1 | 1.03849510 | 0.40467453 | 4.75827411 | 0.018 | 0.03 |

**Table S13.** Pairwise PERMANOVA comparison of the metagenomic composition of Archaea across ecosystems and tissue. P-values were adjusted for multiple testing using the Benjamini–Hochberg false discovery rate (FDR) correction. Df: degrees of freedom; Sum of Sqs: sum of squares; R²: proportion of variance explained; F: pseudo-F statistic; P value: uncorrected p-value; P adj: FDR-adjusted p-value.

| ***Comparison by ecosystem*** | Df | Sum Of Sqs | R^2^ | F | *P* value | *P* adj |
| --- | --- | --- | --- | --- | --- | --- |
| Freshwater vs Seawater | 1 | 0.39277354 | 0.07944 | 2.41629 | 0.02 | 0.06 |
| Freshwater vs Terrestrial | 1 | 0.17920323 | 0.17032 | 2.05289 | 0.045 | 0.0675 |
| Seawater vs Terrestrial | 1 | 0.21402150 | 0.04879 | 1.12834 | 0.199 | 0.199 |
| ***Comparison by tissue*** | Df | Sum Of Sqs | R^2^ | F | *P* value | *P* adj |
| Digestive gland vs Foot | 1 | 0.19878943 | 0.24559511 | 2.60438515 | 0.005 | 0.035 |
| Foot vs Gut | 1 | 0.29111139 | 0.34453645 | 4.20510273 | 0.007 | 0.035 |
| Foot vs Whole body | 1 | 0.17435381 | 0.33212135 | 4.47550192 | 0.004 | 0.035 |
| Foot vs Gill | 1 | 0.56970657 | 0.17404856 | 3.37159896 | 0.012 | 0.039 |
| Gut vs Whole body | 1 | 0.30243147 | 0.34836328 | 3.74218167 | 0.013 | 0.039 |
| Gill vs Whole body | 1 | 0.52517784 | 0.16206059 | 2.90105555 | 0.03 | 0.075 |
| Body wall vs Foot | 1 | 0.15628007 | 0.42400078 | 4.41668070 | 0.04 | 0.07833333 |
| Digestive gland vs Gut | 1 | 0.24276341 | 0.22720123 | 1.76398756 | 0.046 | 0.07833333 |
| Gill vs Gut | 1 | 0.44870312 | 0.13325069 | 2.15230594 | 0.047 | 0.07833333 |
| Body wall vs Whole body | 1 | 0.13663443 | 0.37866500 | 3.04718878 | 0.056 | 0.07909091 |
| Digestive gland vs Gill | 1 | 0.38553886 | 0.11470953 | 1.81401867 | 0.058 | 0.07909091 |
| Digestive gland vs Whole body | 1 | 0.11693388 | 0.15813481 | 1.31487043 | 0.07 | 0.0875 |
| Body wall vs Gill | 1 | 0.29503145 | 0.10272081 | 1.37376390 | 0.136 | 0.14571429 |
| Body wall vs Gut | 1 | 0.21717884 | 0.33692818 | 2.03252899 | 0.133333 | 0.14571429 |
| Body wall vs Digestive gland | 1 | 0.14276751 | 0.22770717 | 1.17938256 | 0.4 | 0.4 |

**Table S14.** Pairwise PERMANOVA comparison of the metagenomic composition of Bacteria across ecosystems and tissue. P-values were adjusted for multiple testing using the Benjamini–Hochberg false discovery rate (FDR) correction. Df: degrees of freedom; Sum of Sqs: sum of squares; R²: proportion of variance explained; F: pseudo-F statistic; P value: uncorrected p-value; P adj: FDR-adjusted p-value.

| ***Comparison by ecosystem*** | Df | Sum Of Sqs | R^2^ | F | *P* value | *P* adj |
| --- | --- | --- | --- | --- | --- | --- |
| Freshwater vs Seawater | 1 | 0.61738796 | 0.08502740 | 2.50908029 | 0.009 | 0.027 |
| Freshwater vs Terrestrial | 1 | 0.34523399 | 0.15616818 | 1.66563243 | 0.068 | 0.102 |
| Seawater vs Terrestrial | 1 | 0.31800008 | 0.05103374 | 1.18312133 | 0.235 | 0.235 |
| ***Comparison by tissue*** | Df | Sum Of Sqs | R^2^ | F | *P* value | *P* adj |
| Gill vs Whole body | 1 | 0.93462884 | 0.21302374 | 4.06029532 | 0.001 | 0.015 |
| Digestive gland vs Foot | 1 | 0.21790979 | 0.22532760 | 2.03607771 | 0.012 | 0.0325 |
| Foot vs Gut | 1 | 0.92223451 | 0.48254408 | 6.52772240 | 0.013 | 0.0325 |
| Foot vs Whole body | 1 | 0.32285071 | 0.36496782 | 4.59778676 | 0.009 | 0.0325 |
| Gill vs Gut | 1 | 0.97913552 | 0.20150327 | 3.53294589 | 0.01 | 0.0325 |
| Gut vs Whole body | 1 | 1.01329250 | 0.45494985 | 5.84285481 | 0.013 | 0.0325 |
| Foot vs Gill | 1 | 0.76882644 | 0.19236889 | 3.57283571 | 0.019 | 0.04071429 |
| Digestive gland vs Gut | 1 | 0.76374371 | 0.35274670 | 3.26994120 | 0.022 | 0.04125 |
| Body wall vs Foot | 1 | 0.14555396 | 0.42457443 | 3.68922104 | 0.056 | 0.09 |
| Digestive gland vs Gill | 1 | 0.59253573 | 0.13998779 | 2.27883872 | 0.06 | 0.09 |
| Body wall vs Gut | 1 | 0.63363000 | 0.42722804 | 2.98358205 | 0.06666667 | 0.09090909 |
| Digestive gland vs Whole body | 1 | 0.24274543 | 0.19947407 | 1.74425138 | 0.089 | 0.11125 |
| Body wall vs Gill | 1 | 0.44712560 | 0.12646900 | 1.73734875 | 0.106 | 0.12230769 |
| Body wall vs Whole body | 1 | 0.20732256 | 0.32929065 | 2.45479391 | 0.154 | 0.165 |
| Body wall vs Digestive gland | 1 | 0.15026658 | 0.19772754 | 0.98583736 | 0.4 | 0.4 |

**Table S15.** Pairwise PERMANOVA comparison of the metagenomic composition of Fungi across ecosystems and tissue. P-values were adjusted for multiple testing using the Benjamini–Hochberg false discovery rate (FDR) correction. Df: degrees of freedom; Sum of Sqs: sum of squares; R²: proportion of variance explained; F: pseudo-F statistic; P value: uncorrected p-value; P adj: FDR-adjusted p-value.

| ***Comparison by ecosystem*** | Df | Sum Of Sqs | R^2^ | F | *P* value | *P* adj |
| --- | --- | --- | --- | --- | --- | --- |
| Freshwater vs Terrestrial | 1 | 0.06926350 | 0.21529445 | 3.01799696 | 0.004 | 0.012 |
| Freshwater vs Seawater | 1 | 0.13984530 | 0.06700899 | 2.01100721 | 0.039 | 0.0585 |
| Seawater vs Terrestrial | 1 | 0.07072991 | 0.03673690 | 0.87717333 | 0.399 | 0.399 |
| ***Comparison by tissue*** | Df | Sum Of Sqs | R^2^ | F | *P* value | *P* adj |
| Digestive gland vs Foot | 1 | 0.13227776 | 0.24237768 | 2.55935103 | 0.005 | 0.025 |
| Foot vs Whole body | 1 | 0.20968566 | 0.41834742 | 6.47315409 | 0.004 | 0.025 |
| Gill vs Whole body | 1 | 0.26836518 | 0.21778091 | 4.17621308 | 0.004 | 0.025 |
| Foot vs Gut | 1 | 0.09153172 | 0.38355815 | 4.97770427 | 0.007 | 0.02625 |
| Body wall vs Foot | 1 | 0.06911164 | 0.56310786 | 9.02226124 | 0.018 | 0.045 |
| Foot vs Gill | 1 | 0.14341742 | 0.16060739 | 3.06140204 | 0.016 | 0.045 |
| Body wall vs Whole body | 1 | 0.15112325 | 0.36059102 | 3.38366545 | 0.043 | 0.084375 |
| Digestive gland vs Gill | 1 | 0.16436162 | 0.13146799 | 2.11915264 | 0.045 | 0.084375 |
| Body wall vs Gut | 1 | 0.07674424 | 0.38316869 | 3.10594390 | 0.054 | 0.08590909 |
| Digestive gland vs Whole body | 1 | 0.21783942 | 0.25759483 | 2.42881364 | 0.061 | 0.08590909 |
| Gut vs Whole body | 1 | 0.18957447 | 0.34403292 | 3.67126730 | 0.063 | 0.08590909 |
| Digestive gland vs Gut | 1 | 0.10281797 | 0.17539322 | 1.27619530 | 0.106 | 0.12230769 |
| Gill vs Gut | 1 | 0.10082334 | 0.10955508 | 1.72247728 | 0.098 | 0.12230769 |
| Body wall vs Gill | 1 | 0.08505588 | 0.10487198 | 1.52306222 | 0.191 | 0.20464286 |
| Body wall vs Digestive gland | 1 | 0.09836542 | 0.20145468 | 1.26138536 | 0.289 | 0.289 |
